# Evaluation of Time-related Bias With Non-user Control

**DOI:** 10.2188/jea.JE20250126

**Published:** 2026-04-05

**Authors:** Hiroya Morita, Kentaro Matsuura, Nodoka Seya, Masataka Taguri

**Affiliations:** 1 Department of Health Data Science, Tokyo Medical University, Tokyo, Japan; 2 Chugai Pharmaceutical Co., Ltd., Tokyo, Japan

**Keywords:** excluded immortal time bias, misclassified immortal time bias, time-zero, time-varying confounding

## Abstract

**Background:**

In observational studies estimating the association between treatment and time-to-event outcomes, time-related biases can substantially impact results. Immortal time bias is one of such biases, and two types are known: misclassified immortal time bias and excluded immortal time bias. These biases often arise from incorrect time-zero definition, especially with non-user controls. This study aims to illustrate immortal time bias in non-user controls using formulas, simulations, and real-world data.

**Methods:**

For our simulations, we considered two scenarios: one with no confounding and no treatment effect, and the other with time-dependent confounding. We compare three different settings of time-zero for treatment and control groups. Method 1: Both groups were followed from cohort entry date (CED). Method 2: The treatment group was followed from treatment initiation date (TID), while the non-user group was followed from CED. Method 3: The treatment group was followed from TID, and non-users were matched to treatment patients, followed from the corresponding TID of their matched patient.

**Results:**

Our simulation showed that both method 1 and method 2 can exhibit large biases in the estimated treatment effect due to immortal time bias. The magnitude of the bias is greater for method 1 than for method 2. On the other hand, method 3 showed almost no bias. Even in the presence of time-dependent confounding, method 3 did not introduce bias.

**Conclusion:**

To reduce time-related biases, it is crucial for researchers to carefully define an appropriate time-zero, especially when using a non-user control group.

## INTRODUCTION

In recent years, numerous studies have evaluated treatment safety and efficacy using real-world data (RWD).^1^ However, observational studies face challenges, such as confounding,^2^ the validity of outcome definitions,^3^^,^^4^ and prevalent user bias.^5^ One of the most critical biases is time-related bias, which has been shown to affect many studies that report remarkable effectiveness.^6^

Immortal time bias is a type of time-related bias that occurs depending on how time-zero is defined. It introduces periods of immortal time during which no outcomes can occur, leading to biased results. Two types exist: misclassified and excluded immortal time bias.^7^^–^^9^ The former occurs when pre-treatment time is misclassified as treatment time, while the latter excludes pre-treatment time from analysis. While the term “immortal time bias” typically refers to misclassified immortal time bias and is frequently discussed in papers and reviews,^10^ excluded immortal time bias is less well-known, though it is believed to occur in many cases.

Although the use of active comparators is recommended to address confounding by indication,^11^ non-users are often employed as control groups due to specific research questions or the lack of appropriate comparators.^12^ However, determining the correct time-zero for non-users can be challenging and is sometimes done incorrectly. For instance, studies investigating the combination of fenofibrate with statins in patients with high triglyceride levels may be using an incorrect time-zero. One such study reported a hazard ratio (HR) of 0.929 with cardiovascular disease (CVD) events as the outcome.^13^ In this analysis, the fenofibrate treatment group was followed from the start of treatment, while non-user group was followed from the time of eligibility, potentially introducing excluded immortal time bias. Although limited to diabetic patients, randomized controlled trials (RCTs) have shown that the risk of CVD events does not significantly change when statins are combined with fibrates.^14^^,^^15^ In contrast, observational studies may show apparent preventive effects in the treatment group due to excluded immortal time bias.

The importance of correctly defining time-zero is also discussed in the target trial emulation framework proposed by Hernán et al, which discusses the relationship between time-zero, eligibility, and treatment strategy assignment.^16^^–^^18^ This study clarifies that misclassified immortal time bias occurs when time-zero precedes the initiation of the treatment strategy. Additionally, a previous study using the JMDC (formerly Japan Medical Data Center Co., Ltd.) database compared six different time-zero definitions for non-user controls and found that the time-zero associated with two types of immortal time bias appeared to favor the treatment group compared to other approaches.^19^ However, these studies do not provide a quantitative assessment of the degree of bias in various scenarios, including those involving time-varying confounders.^20^ Better understanding of when and how these two types of immortal time bias occur, based on data characteristics, is crucial for analysis.

In this paper, we begin by introducing three ways of setting time-zero: one that leads to misclassified immortal time bias, one that causes excluded immortal time bias, and a correct approach that avoids both types of bias.^21^ We then use mathematical formulas and simulations to examine the biases in these methods and to identify the situations in which a large bias is likely to occur. The simulations were conducted under two scenarios: a simple setting with no confounding and a realistic setting with time-varying confounding. Finally, we apply the methods to RWD.

## EVALUATION OF IMMORTAL TIME BIAS

### Three time-zero settings

**Figure 1.**
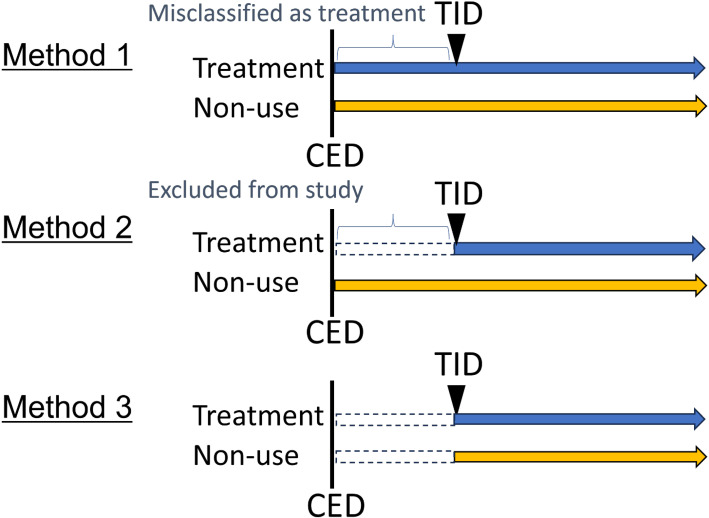
A diagram of the three different time-zero methods. Method 1: both groups are followed from the cohort entry date (CED). Method 2: the treatment group is followed from the treatment initiation date (TID), while the non-user group is followed from CED. Method 3: the treatment group is followed from TID. The non-user patients are matched with the treatment patients in terms of the length from CED to TID. The control group are followed from the corresponding date of TID of their matched treatment patient.

In this study we compare three time-zero settings, Method 1 to 3 (Figure 1). Method 1: Both groups are followed from cohort entry date (CED). Method 2: The treatment group is followed from treatment initiation date (TID) while non-user group is followed from CED. Method 3: The treatment group is followed from TID. The non-user patients are matched with the treatment patients in terms of the length from CED to TID. The control group is followed from the corresponding date of TID of their matched treatment patient.^21^ To avoid selection bias, any individual not receiving treatment at that time point could be matched as a non-user group.^21^ In the next subsection, we will illustrate the biases caused by method 1 and method 2 using the mathematical formulas.

### Evaluation of immortal time bias using the formula

**Figure 2.**
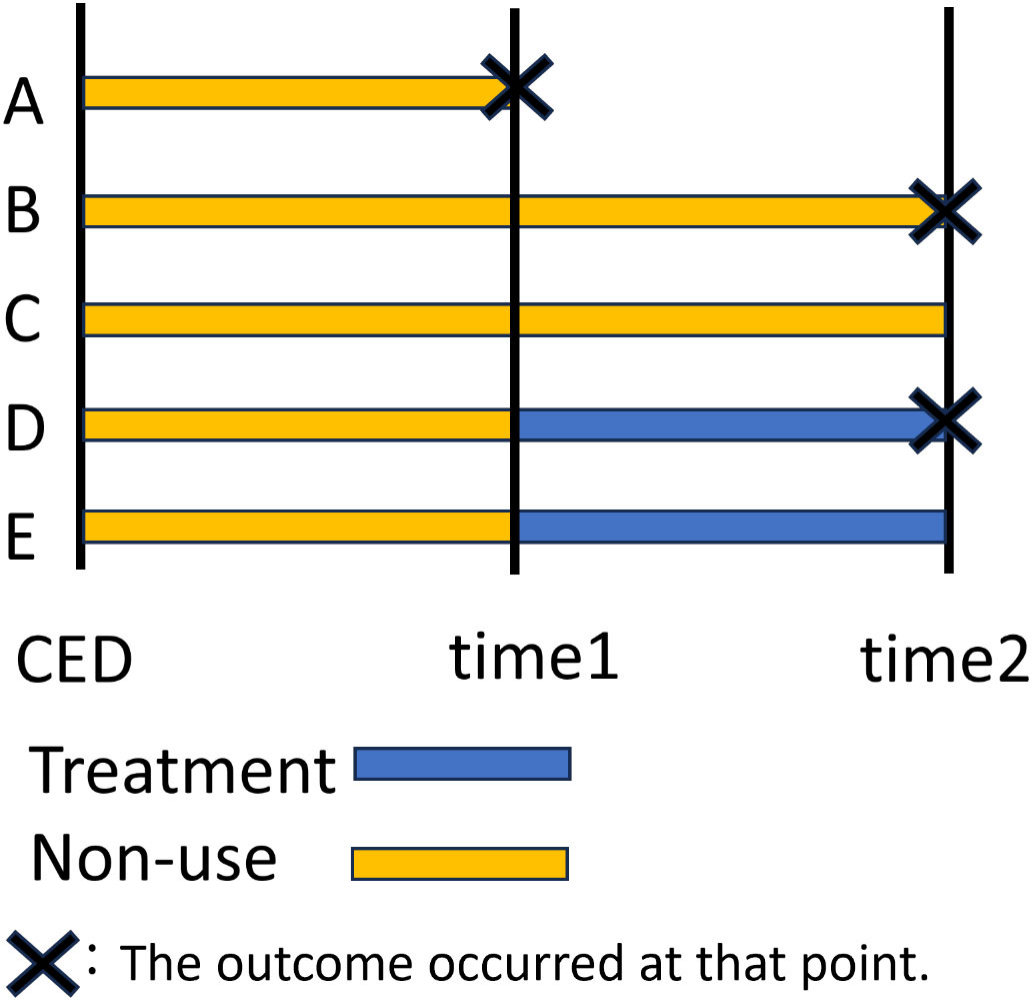
A diagram for the hypothetical scenario with two time points. A: non-user at time point 1 who experienced an outcome at time point 1. B: non-user up to time point 2 who experienced an outcome at time point 2. C: non-user up to time point 2 who did not experience an outcome at time point 2. D: Non-user at time point 1 who initiated treatment at time point 2 and experienced an outcome at time point 2. E: Non-user at time point 1 who initiated treatment at time point 2 and did not experience an outcome at time point 2.

We consider a simple situation with two time points (Figure 2). Here, we describe a simple scenario in which there is no treatment effect and the hazard is constant over time. In eMaterial 1, we consider a more realistic situation in which the baseline hazard changes over time and a treatment effect is present. For simplicity, we assume that the hazard of the event of interest λ is constant for both groups and time points. Based on the patterns of the received treatment and outcome occurrence, we classified individuals into five patterns. A–E represent patterns: A, B, and C involve non-users at time points 1 and 2, with different outcome occurrences, while D and E involve treatment initiation at time point 2, with or without outcomes.

Let the probabilities of existence of five types of A to E be denoted as Pr(A), Pr(B), Pr(C), Pr(D), and Pr(E), respectively. Given that there is no treatment group at time point 1 in this situation, λ can be expressed as the following three equalities:

$$\begin{array}{rl} \lambda & =\frac{\mathrm{Pr}(\text{A})}{\mathrm{Pr}(\text{A})+\mathrm{Pr}(\text{B})+\mathrm{Pr}(\text{C})+\mathrm{Pr}(\text{D})+\mathrm{Pr}(\text{E})}\\ & =\frac{\mathrm{Pr}(\text{B})}{\mathrm{Pr}(\text{B})+\mathrm{Pr}(\text{C})}=\frac{\mathrm{Pr}(\text{D})}{\mathrm{Pr}(\text{D})+\mathrm{Pr}(\text{E})} \end{array}$$ (1)

where Pr(A) + Pr(B) + Pr(C) + Pr(D) + Pr(E) = 1. These three equalities hold under the assumption that hazards remain constant across time points and treatments.

Method 1 uses a control group consisting of A, B, and C and a treatment group consisting of D and E, with time-zero set at CED. In method 2, the same groups are used as in method 1; however, the treatment group’s time-zero is set at time point 1 instead of CED. Finally, method 3 uses a control group of B and C and a treatment group of D and E, with time-zero for both groups set at time point 1.

For method 1, let $\lambda_{1\text{trt}}^{\text{Method}\;1}$ be the hazard for the treatment group at time point 1, and $\lambda_{1\text{non}}^{\text{Method}\;1}$ be the hazard for the non-user group at time point 1. For method 2, let $\lambda_{2\text{trt}}^{\text{Method}\;2}$ be the hazard for the treatment group at time point 2, and $\lambda_{1\text{non}}^{\text{Method}\;2}$ be the hazard for the non-user group at time point 1. For method 3, let $\lambda_{2\text{trt}}^{\text{Method}\;3}$ be the hazard for the treatment group at time point 2, and $\lambda_{2\text{non}}^{\text{Method}\;3}$ be the hazard for the non-user group at time point 2.

For method 1, the hazards are:

$$\lambda_{\text{1trt}}^{\text{Method 1}}=\frac{0}{\mathrm{Pr}(\text{D})+\mathrm{Pr}(\text{E})}=0$$ (2)

$$\begin{array}{rl} \lambda_{\text{1non}}^{\text{Method 1}} & =\frac{\mathrm{Pr}(\text{A})}{\mathrm{Pr}(\text{A})+\mathrm{Pr}(\text{B})+\mathrm{Pr}(\text{C})}\\ & \;>\frac{\mathrm{Pr}(\text{A})}{\mathrm{Pr}(\text{A})+\mathrm{Pr}(\text{B})+\mathrm{Pr}(\text{C})+\mathrm{Pr}(\text{D})+\mathrm{Pr}(\text{E})}=\lambda \end{array}$$ (3)

Method 1 induces misclassified immortal time bias, resulting in a hazard $\lambda_{1\text{trt}}^{\text{Method}\;1}=0$ , indicating a period during which no events occur. Additionally, the hazard $\lambda_{1\text{non}}^{\text{Method}\;1}$ is larger than the true hazard λ. These factors lead to a bias that underestimates the true HR. In this case, the estimated HR is zero.

For method 2, the hazards are:

$$\lambda_{\text{2trt}}^{\text{Method 2}}=\frac{\mathrm{Pr}(\text{D})}{\mathrm{Pr}(\text{D})+\mathrm{Pr}(\text{E})}=\lambda$$ (4)

$$\begin{array}{rl} \lambda_{\text{1non}}^{\text{Method 2}} & =\frac{\mathrm{Pr}(\text{A})}{\mathrm{Pr}(\text{A})+\mathrm{Pr}(\text{B})+\mathrm{Pr}(\text{C})}\\ & \;>\frac{\mathrm{Pr}(\text{A})}{\mathrm{Pr}(\text{A})+\mathrm{Pr}(\text{B})+\mathrm{Pr}(\text{C})+\mathrm{Pr}(\text{D})+\mathrm{Pr}(\text{E})}=\lambda \end{array}$$ (5)

Method 2 is subject to excluded immortal time bias, which leads to an overestimation of the hazard $\lambda_{1\text{non}}^{\text{Method}\;2}$ in the non-user group. The magnitude of bias increases as the proportion of subjects who switch to the treatment increases, which is represented by the sum of the probabilities Pr(D) and Pr(E). In method 2, when the proportion of subjects switching to the treatment is small, the bias will be minimal. However, in method 1, the hazard for the treatment group becomes zero, so substantial bias occurs even with a small proportion of subjects who switch to the treatment.

For method 3, the hazards are:

$$\lambda_{\text{2trt}}^{\text{Method 3}}=\frac{\mathrm{Pr}(\text{D})}{\mathrm{Pr}(\text{D})+\mathrm{Pr}(\text{E})}=\lambda$$ (6)

$$\lambda_{\text{2non}}^{\text{Method 3}}=\frac{\mathrm{Pr}(\text{B})}{\mathrm{Pr}(\text{B})+\mathrm{Pr}(\text{C})}=\lambda$$ (7)

Method 3 is the appropriate method, as $\lambda_{2\text{trt}}^{\text{Method}\;3}$ and $\lambda_{2\text{non}}^{\text{Method}\;3}$ correctly equal to the true hazard λ.

Misclassified immortal time bias is relatively easy to understand, as the hazard for the treatment group is zero. However, excluded immortal time bias is more complex, so we conducted a simple numerical examination to illustrate its impact. Specifically, we examine the HR given using method 2, $\lambda_{2\text{trt}}^{\text{Method}\;2}/\lambda_{1\text{non}}^{\text{Method}\;2}$ , under the values of Pr(A) and {Pr(D) + Pr(E)}. The HRs at time point 1 for method 2 are shown in Table 1. The hazard in the non-user group at time point 1 increases as the proportions of groups D and E increase, resulting in an underestimation of the HR. As demonstrated by the inequality (5) and Table 1, the proportion of subjects who switch from the control to the treatment (that is, starting treatment) affects the magnitude of excluded immortal time bias.

**Table 1.** The result of a numerical examination evaluating the excluded immortal time bias

Proportion of D + E	Proportion of A								

	0.1	0.2	0.3	0.4	0.5	0.6	0.7	0.8	0.9
0.1	0.9	0.9	0.9	0.9	0.9	0.9	0.9	0.9	0.9
0.2	0.8	0.8	0.8	0.8	0.8	0.8	0.8	0.8	NA
0.3	0.7	0.7	0.7	0.7	0.7	0.7	0.7	NA	NA
0.4	0.6	0.6	0.6	0.6	0.6	0.6	NA	NA	NA
0.5	0.5	0.5	0.5	0.5	0.5	NA	NA	NA	NA
0.6	0.4	0.4	0.4	0.4	NA	NA	NA	NA	NA
0.7	0.3	0.3	0.3	NA	NA	NA	NA	NA	NA
0.8	0.2	0.2	NA	NA	NA	NA	NA	NA	NA
0.9	0.1	NA	NA	NA	NA	NA	NA	NA	NA

This table presents the hazard ratios of method 2 according to the values of the proportion of A and that of D + E. Each cell shows the hazard ratio $\lambda_{2\text{trt}}^{\text{Mehod}\;2}/\lambda_{1\text{non}}^{\text{Method}\;2}$ obtained using method 2. The true hazard ratio is 1. The columns indicate the proportion of A, which corresponds to the true hazard. The rows represent the proportion of individuals switching to the treatment at time point 2, denoted as D + E. Since the sum of A + D + E cannot exceed 1, impossible combinations are marked as not applicable (NA).

In eMaterial 1, we considered a scenario in which the treatment had a non-zero effect and the baseline hazard varied over time. As in the simpler setting, immortal time bias still occurs, but a key difference was that method 2 introduced additional bias due to misalignment in the time-zero definition with respect to time since cohort entry. The direction of this bias may vary in either direction. This bias represents time lag and latency bias, which is a type of time-related bias.^19^^,^^22^

## SIMULATION STUDY

Here we conduct a simulation study to quantify the magnitudes of bias by Methods 1 and 2 under some specific conditions.

### Setting

The sample size is set at *n* = 10,000 with two time points and the simulation is repeated 1,000 times. For simplicity, we consider a scenario with no confounding and no treatment effect. At time point *t* (*t* = 1,2), treatment *A_t_* is generated as follows: if *A*_1_ = 1, then *A*_2_ = 1, otherwise logit(Pr(*A*_1_ = 1)) = logit(Pr(*A*_2_ = 1|*A*_1_ = 0,*Y*_1_ = 0)) = β_1_. In other words, it reflects a situation where treatment is continued once initiated. Similarly, the outcome *Y_t_* at time point t is generated as logit(Pr(*Y*_1_ = 1|*A*_1_)) = logit(Pr(*Y*_2_ = 1|*A*_1_,*A*_2_,*Y*_1_ = 0)) = γ_1_. In this study, three different values of β_1_ were used: −1, −2, and −3, while γ_1_ was set to −1. The probability of switching to treatment remains constant across time points (27%, 12%, 5%), and the probability of outcome occurring is also constant across time points (27%). A more realistic scenario with time-varying confounding is described in eMaterial 2. In this scenario with time-varying confounding, we assumed no treatment effect and constant HR throughout the study period.

### Statistical analysis

We compare three time-zero settings: methods 1, 2, and 3. Follow-up is conducted from time-zero to the occurrence of the outcome or the end of the study. At time zero for each method, 1:1 random matching was performed, with treated individuals used as the reference group. We estimate the HR using a Cox regression model with treatment as a covariate.

### Simulation results

**Figure 3.**
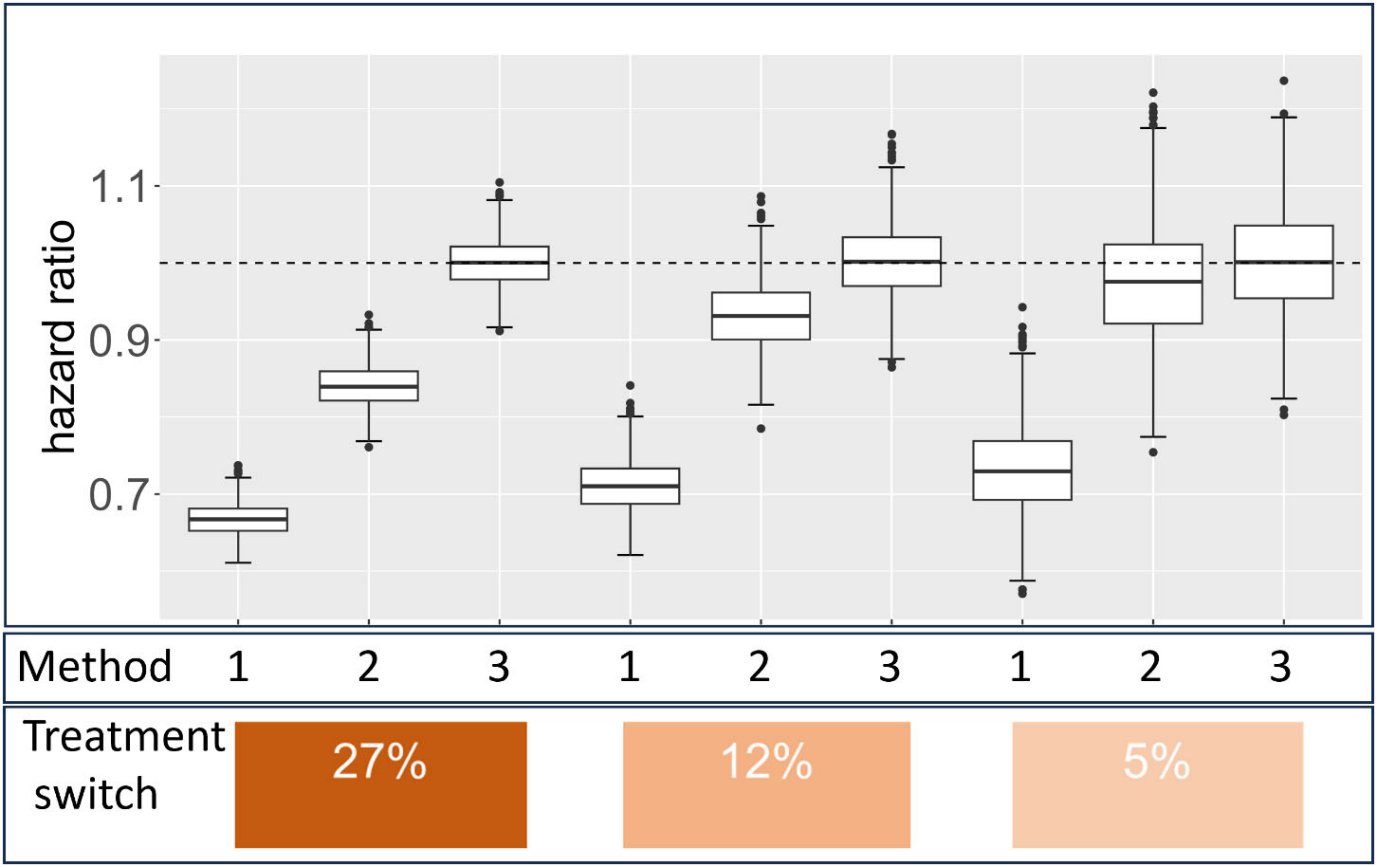
Simulation results comparing methods 1, 2, and 3. The figure shows box plots of estimated HRs over 1,000 simulation runs, with the true value set at 1. The probabilities of receiving treatment at each time point are set at 27%, 12%, and 5%, respectively.

Figure 3 shows the simulation results. When comparing the three time-zero settings with non-users as the control group, we found that in method 1, the HR estimates showed a negative bias from the true value of 1, leading to an overestimation of the treatment effect. Similarly, in method 2, the HR estimates also showed a negative bias from the true value. Notably, the bias was larger when the proportion of individuals switching to the treatment was high, as expected from the examinations described previously. Method 2 showed less bias when the proportion of switching to the treatment was small, whereas method 1 exhibited a large bias even in such situations, consistent with the results derived from the formula. In contrast, method 3 yielded nearly unbiased HR estimates. In the above setting where time-dependent confounding does not exist, the impact of immortal time bias can be removed with a time-dependent Cox regression without matching. However, in the other simulation where time-varying confounders exist, adjusting for time-varying confounders using Cox regression resulted in bias due to time-varying confounding, whereas method 3 showed almost no bias (eFigure 1, eFigure 2 and eFigure 3).

## METHODS

### Data sources

We used data from JMDC Inc., one of the most commonly used databases in Japan. Comprehensive details regarding this database are provided elsewhere.^19^^,^^23^

### Study design and population

We used data from 2011 to 2023, focusing on individuals who had been prescribed statins. For individuals using statins, the CED was defined as the first date their triglyceride (TG) levels exceeded 150 mg/dL. Individuals who had experienced an outcome prior to the CED were excluded from the analysis. Among the selected participants, 3,366 had used fenofibrate or bezafibrate at least once after their CED, while 144,752 had never used fenofibrate or bezafibrate. Follow-up for the study continued until December 2023.

### Measurements and definitions

Adjustments were made with reference to the factors considered in previous studies.^13^ High-density lipoprotein (HDL) cholesterol, low-density lipoprotein (LDL) cholesterol, and TGs were measured using blood test results. Natural logarithm transformations were applied to HDL cholesterol, LDL cholesterol, and TGs. Obesity was defined as a body mass index (BMI) of 25 kg/m^2^ or higher. Hypertension was defined as either a blood pressure reading of 140/90 mm Hg or the use of blood pressure-lowering medications. Diabetes was identified based on the use of diabetes medications. Additional adjustment factors included age, gender, smoking status, history of renal failure or dialysis as reported in interviews, and histories of cerebrovascular disease or cardiovascular disease also reported in interviews. The balance of covariates was assessed using standardized mean differences (SMD).

### Study outcomes and follow-up

The outcome of the study were CVD events, with outcome definitions consistent with those used in previous studies.^13^ Myocardial infarction was identified using International Classification of Diseases, 10^th^ revision (ICD-10) codes I21 or I22, and ischemic stroke was identified using ICD-10 codes I63 or I64. Follow-up began from the time-zero and continued until the occurrence of an event or the end of the follow-up period.

### Statistical analyses

As in the simulation, we analyzed three time-zero settings: methods 1 to 3. We conducted two types of analyses: one in which covariates were adjusted using propensity score matching and one in which covariates were unadjusted with random matching. Propensity score matching with treated individuals used as the reference group was performed at time-zero for each method. The propensity scores were estimated using logistic regression with linear terms of the covariates described previously. For method 3, exposure sets were assessed for every month after CED and propensity scores were estimated in each exposure sets. The matching was conducted using 1:1 nearest neighbor matching with a caliper of 0.2. HRs and 95% confidence intervals (CIs) were calculated using Cox regression models, with the treatment as the explanatory variable. All analyses were performed according to the intention-to-treat principle, indicating that changes in treatment after matching were not considered in the analysis. In method 3, since the same individual could be matched multiple times, robust variance estimation was applied with clustering at the individual level.^24^^,^^25^

## RESULTS

**Figure 4.**
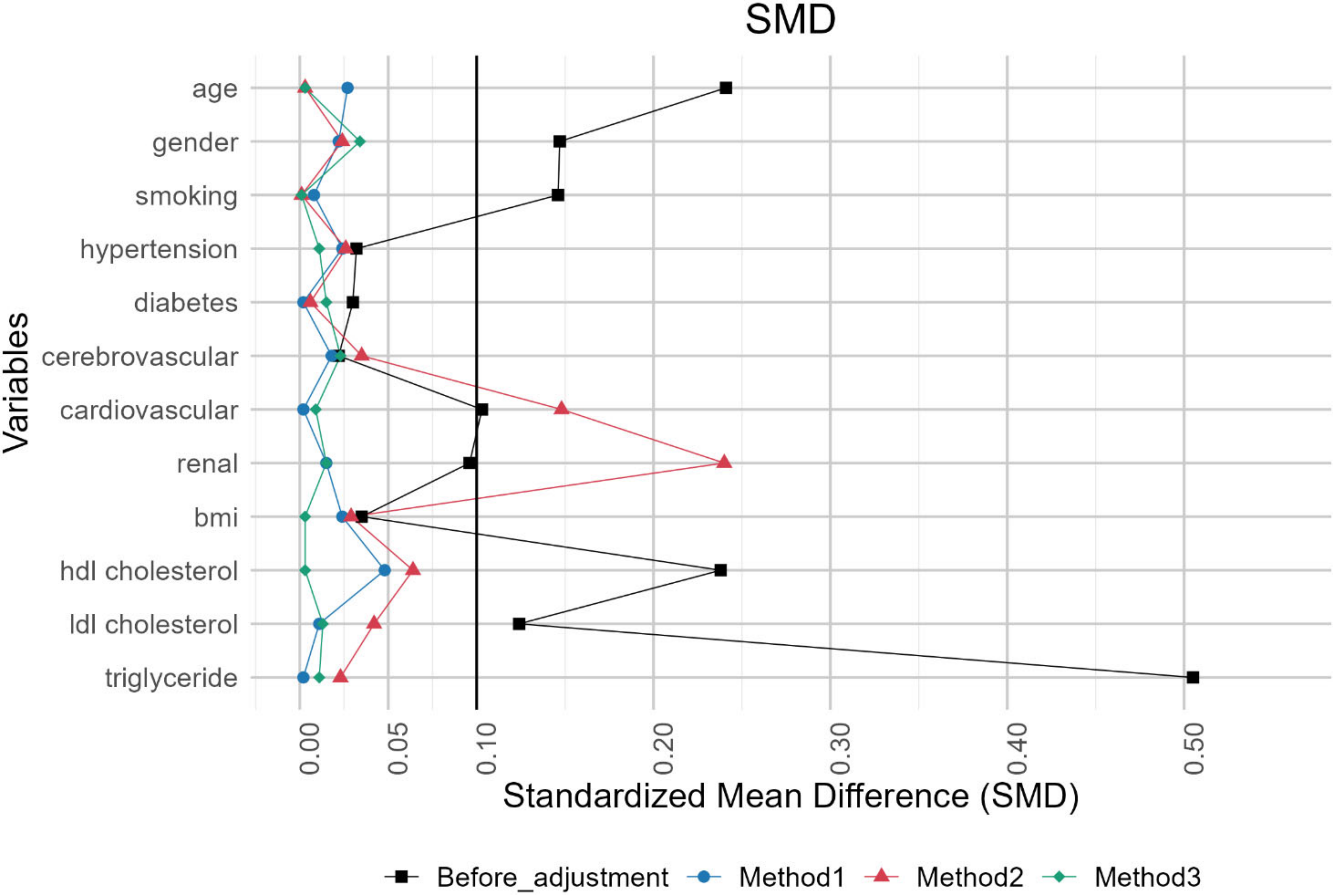
Absolute value of the standardized mean differences (SMDs) in the RWD analysis. Figure presents the absolute value of SMD, comparing the results before adjustment with those obtained using methods 1, 2, and 3. Each method employs a 1:1 propensity score matching at the corresponding time-zero. A horizontal line is drawn at the reference value of 0.1 for the absolute value of SMD.

After applying the eligibility and exclusion criteria, the target population consisted of 148,118 individuals. Table 2 presents the baseline covariate values at CED. Figure 4 illustrates the SMD before and after adjustment. Although the timing of matching differed across methods, we only reported the unadjusted SMD at the CED, which corresponds to the pre-adjustment time point in method 1.

**Table 2.** Baseline characteristics of the RWD analysis

Variable	Only statin *N* = 144,752	Combination of statin and fibrate *N* = 3,366	SMD
Age, years	54.6 (8.8)	52.6 (8.3)	0.241
Male	106,229 (73.4)	2,680 (79.6)	0.147
Smoking	36,341 (25.1)	1,066 (31.7)	0.146
Hypertension	80,105 (55.3)	1,809 (53.7)	0.032
Diabetes	27,040 (18.7)	590 (17.5)	0.03
Medical history of cerebrovascular	2,133 (1.5)	41 (1.2)	0.022
Medical history of cardiovascular	9,029 (6.2)	134 (4.0)	0.103
Medical history of renal	1,889 (1.3)	14 (0.4)	0.096
BMI	26.1 (4.1)	26.2 (3.9)	0.035
HDL cholesterol	54.0 (12.3)	51.0 (12.6)	0.238
LDL cholesterol	126.2 (35.9)	121.6 (37.8)	0.124
Triglyceride	201.7 (79.7)	266.1 (161.7)	0.505

RWD, real-world data; SMD, standardized mean difference.

The table presents the baseline covariates for the statin group and the combined statin-fibrate group, along with the absolute value of the SMD. Categorical variables are reported as the number of individuals (%), while continuous variables are shown as the mean (standard deviation).

Before matching, some variables were not balanced, and larger SMDs were observed. Although some variables in method 2 still showed imbalances, overall, the SMD remained below 0.1, indicating that good balances were achieved.

Table 3 shows the results of the three time-zero analyses, including both the unadjusted results and adjusted for covariates. In method 1, a substantial treatment effect was appeared to be observed, with a HR of 0.83 (95% CI, 0.71–0.96). However, in method 2, the HR was 1.14 (95% CI, 0.99–1.31). In contrast, in method 3, the HR was 1.18 (95% CI, 1.03–1.36), indicating a treatment effect in favor of the non-user group. A naive analysis without adjusting for covariates yielded almost identical results.

**Table 3.** Estimation results of the RWD analysis

Method	Hazard ratio (95% CI) unadjusted	Hazard ratio (95% CI) adjusted
Method 1	0.71 (0.62–0.82)	0.83 (0.71–0.96)
Method 2	1.19 (1.03–1.37)	1.13 (0.99–1.31)
Method 3	1.30 (1.12–1.50)	1.18 (1.03–1.36)

CI, confidence interval; RWD, real-world data.

This table presents the estimation results of the hazard ratios and their 95% CI obtained using method 1 to 3. The table includes two sets of results: unadjusted, which involved random matching without covariate adjustment, and adjusted, which applied propensity score matching.

## DISCUSSION

The simulation and RWD analysis conducted in this study confirmed that bias can arise depending on how time-zero is defined when non-users are used as the control group. Consistent with previous studies, our analysis confirmed that immortal time bias tends to overestimate treatment effectiveness and underestimate adverse events.^10^

In this study, immortal time bias was systematically examined using mathematical formulas. By applying these formulas, we demonstrated that the magnitude of bias increases as the proportion of subjects who switch to the treatment increases in the case that excluded immortal time bias occurs. Additionally, we showed that the degree of bias is more pronounced for misclassified immortal time bias compared to excluded immortal time bias. The simulation study and RWD analyses were employed to evaluate the biases outlined through the formulas. The simulation results indicated that using the two incorrect time-zero settings led to biased estimates, with the bias being larger for the misclassified immortal time bias. Similarly, RWD analysis demonstrated that applying the incorrect method shifted the estimates in favor of the treatment group, regardless of whether confounding adjustments were made. It is known that the proportion of the treatment group affects the magnitude of misclassified immortal time bias.^26^ In this study, we not only corroborated these findings but also demonstrated that the proportion of switching to treatment substantially affects the extent of the excluded immortal time bias. However, when the baseline hazard varies over time, time lag and latency bias may arise,^19^^,^^22^ and the resulting bias may not follow the same pattern (eMaterial 1).

Generally, one approach to address immortal time bias is to analyze it as a time-dependent exposure.^27^ Simulations have shown that such an analysis using a time-dependent Cox regression can eliminate the impact of immortal time bias.^28^ However, in situations where time-varying confounding is present, using time-dependent Cox regression adjusting for time-varying confounders can introduce bias.^29^ This bias, introduced by conditioning on time-dependent confounders in a regression model, arises because such adjustment may lead to collider stratification bias and, in addition, introduce bias by adjusting for variables that also act as mediators of the treatment effects.^20^^,^^29^^,^^30^ Our simulation reveals that method 3 allows for nearly unbiased estimation (eFigure 1, eFigure 2, and eFigure 3).

Several methods have been proposed to address immortal time bias, including landmark analysis and exposure density sampling.^25^^,^^31^^–^^33^ Landmark analysis can provide unbiased estimates under the null hypothesis of no treatment effect; however, in the presence of a true treatment effect, it may introduce bias.^32^ Although method 3 used in this study shares similarities with landmark analysis, a key distinction is that method 3 can address prevalent user bias, whereas landmark analysis may be susceptible to it.^18^^,^^21^ However, in the landmark approach, it can be slightly modified to select only incident users, in which case it is no longer susceptible to such bias. Furthermore, the approach based on exposure density sampling essentially implements the same procedure as method 3.^25^

Method 3 in this study addresses immortal time bias by matching the treatment and control groups based on confounder distribution and treatment initiation timing. Method 3 selects control subjects with similar propensity scores to the treated, thereby estimating the average treatment effect on the treated.^34^ In contrast to other approaches such as the inverse probability weighted time-dependent Cox model (Cox IPW), which can define time-zero either as time since CED^29^ or as time since the TID,^35^ method 3 defines time-zero exclusively as time since TID. In addition, Cox IPW models marginal distribution of potential outcomes and typically targets the average treatment effect on the entire population. When the treatment effect is non-null and the baseline hazard is time-varying, these methods generally target different estimands. As noted above, various methods, including method 3, have been proposed to address immortal time bias; however, it should be noted that even when these (like method 3) target estimation of HRs, the corresponding (interpretation of the) estimands being targeted generally differs across methods, and the assumptions required for valid estimation are not identical. In this paper, the formulation of immortal time bias is based on method 3 and is, therefore, not directly applicable to more general estimands; for example, the formulation would differ if the estimand were that targeted by a Cox IPW. A detailed discussion of these differences is left for future research, but they should be carefully considered when choosing a method. Moreover, while this paper has focused on the HR, there are related works addressing immortal time bias that focus on different measures, such as the restricted mean survival time.^36^^,^^37^

In method 3 of our RWD analysis, the HR exceeded 1, favoring the control group. However, it is improbable that the actual use of fibrates in combination with statins increases the risk of CVD events. The result may be attributed to the differential statin discontinuation rates between the groups. Indeed, the statin discontinuation rate in the fibrate combination group (0.056 per person-month) was about twice as high as that in the statin-only group (0.025 per person-month).

Time-zero is also a crucial factor for the target trial emulation framework, where aligning time-zero with the timing of assessment of eligibility criteria and treatment assignment is considered to be essential.^16^^,^^17^ In the case of excluded immortal time bias, although time-zero, eligibility, and treatment assignment may appear to be aligned within each group, the timing of assessment of eligibility criteria differ between groups. Therefore, it is necessary to ensure that time-zero, eligibility, and treatment assignment are consistently aligned across the entire cohort, not just within each group. Method 3 in this study is satisfied with such criteria.^38^ Another appropriate method is the multiple successive cohorts method (also known as the sequential trial approach), which is conducted as a form of target trial emulation.^39^ While both approaches aim to emulate an RCT for causal inference in observational studies and use a similar time-zero, they differ in the choice of control groups: method 3 uses matched controls with similar propensity scores to the treated, whereas the successive cohorts method uses all individuals eligible at that time as controls, which may also lead to differences in statistical power.^38^^,^^39^

As a limitation of this study, the mathematical formulation was restricted to two time points, although in practice, treatment switching may occur at time points beyond the second. This possibility was not accounted for in the analysis. In addition, the model assumes proportional hazards, and the extent of bias when this assumption is violated remains unclear. In this study, 1:1 matching was employed across all methods; however, using 1:k (k > 1) matching may allow for more efficient estimation. Moreover, while this study evaluated time-related biases in estimating the ITT effect using RWD, different results may arise if a per-protocol effect is estimated instead.

In conclusion, properly defining time-zero is essential to avoid immortal time bias when using non-users as a control group.

## SUPPLEMENTARY MATERIAL

eMaterial 1. Evaluation of bias under more realistic conditions

eMaterial 2. Simulation study in the presence of time-varying confounding

eFigure 1. Simulation results comparing methods 1, 2, 3 and time-dependent Cox regressions adjusted for time-varying confounders. The figure shows box plots of estimated hazard ratios over 1,000 simulation runs, with the true value set at 1. The average proportion of individuals receiving treatment at each time point was approximately 2.7% or 0.4%, while the proportion developing the outcome was approximately 2%.

eFigure 2. Simulation results comparing methods 1, 2, 3 and time-dependent Cox regressions adjusted for time-varying confounders. The figure shows box plots of estimated hazard ratios over 1,000 simulation runs, with the true value set at 1. The average proportion of individuals receiving treatment at each time point was approximately 2.7%, while the proportion developing the outcome was approximately 2%.

eFigure 3. Simulation results of Cox regression without time-varying confounders. The figure shows box plots of estimated hazard ratios over 1,000 simulation runs, with the true value set at 1. The probabilities of receiving treatment at each time point are set at 27%.
