# Supplementary material for: Evaluation of Time-related Bias With Non-user Control
Source: J Epidemiol. 2026 Apr 5;36(4):140–7. doi: 10.2188/jea.JE20250126 (PMC12975769; doi:10.2188/jea.JE20250126)
Supplement: Supplementary file 1 [file je-36-140-s001.pdf]

eMaterial 1. Evaluation of bias under more realistic conditions

We consider a simple situation with two time points (**Figure 2**). Here, we consider a more realistic situation in which the baseline hazard changes over time and a treatment effect is present. Based on the patterns of the received treatment and outcome occurrence, we classified individuals into five patterns. A through E are defined as follows. A: Non-user at time point 1 who experienced an outcome at time point 1. B: Non-user up to time point 2 who experienced an outcome at time point 2. C: Non-user up to time point 2 who did not experience an outcome at time point 2. D: Non-user at time point 1 who initiated treatment at time point 2 and experienced an outcome at time point 2. E: Non-user at time point 1 who initiated treatment at time point 2 and did not experience an outcome at time point 2.

Let the probabilities of the five types A to E be denoted as Pr(A), Pr(B), Pr(C), Pr(D), and Pr(E), respectively. Given that there is no treatment group at time point 1 in this context, we consider three hazards: the hazard for the untreated group at time point 1 (denoted as λ_1), the hazard for the untreated group at time point 2 (λ_2), and the hazard for the treated group at time point 2 (λ_3). Assuming that the baseline hazard changes over time but the hazard ratio remains constant, λ_1 through λ_3 can be expressed as follows:

$$\lambda_1 = h(1) = \frac{\Pr(A)}{\Pr(A) + \Pr(B) + \Pr(C) + \Pr(D) + \Pr(E)} \tag{1}$$

$$\lambda_2 = h(2) = \frac{\Pr(B)}{\Pr(B) + \Pr(C)} \tag{2}$$

$$\lambda_3 = h(2)\exp(\beta) = \frac{\Pr(D)}{\Pr(D) + \Pr(E)} \tag{3}$$

where h(1) and h(2) represent the baseline hazards at each time point, and $\exp(\beta)$ denotes the hazard ratio at the corresponding time.

Method 1 uses a control group consisting of A, B, and C and a treatment group consisting of D and E, with time-zero set at CED. In method 2, the same groups are used as in method 1; however, the treatment group's time-zero is set at time point 1 instead of CED. Finally, method 3 uses a control group of B and C and a treatment group of D and E, with time-zero for both groups set at time point 1.

For method 1, let $\lambda_{1trt}^{Method\,1}$ be the hazard for the treatment group at time point 1, and $\lambda_{1non}^{Method\,1}$ be the hazard for the non-user group at time point 1. For method 2, let $\lambda_{2trt}^{Method\,2}$ be the hazard for the treatment group at time point 2, and $\lambda_{1non}^{Method\,2}$ be the hazard for the non-user group at time point 1. For method 3, let $\lambda_{2trt}^{Method\,3}$ be the hazard for the treatment group at time point 2, and $\lambda_{2non}^{Method\,3}$ be the hazard for the non-user group at time point 2.

For method 1, the hazards are:

$$\lambda_{1trt}^{Method\,1} = \frac{0}{\Pr(\mathrm{D}) + \Pr(\mathrm{E})} = 0 \tag{4}$$

$$\lambda_{1non}^{Method\,1} = \frac{\Pr(\mathrm{A})}{\Pr(\mathrm{A}) + \Pr(\mathrm{B}) + \Pr(\mathrm{C})} > \frac{\Pr(\mathrm{A})}{\Pr(\mathrm{A}) + \Pr(\mathrm{B}) + \Pr(\mathrm{C}) + \Pr(\mathrm{D}) + \Pr(\mathrm{E})} = \lambda_1 \tag{5}$$

Method 1 induces misclassified immortal time bias, resulting in a hazard $\lambda_{1trt}^{Method\,1} = 0$, indicating a period during which no events occur. Additionally, the hazard $\lambda_{1non}^{Method\,1}$ is larger than the true hazard λ_1. These factors lead to a bias that underestimate the true hazard ratio (hazard ratio becomes zero).

For method 2, the hazards are:

$$\lambda_{2trt}^{Method\,2} = \frac{\Pr(\mathrm{D})}{\Pr(\mathrm{D}) + \Pr(\mathrm{E})} = \lambda_3 \tag{6}$$

$$\lambda_{1non}^{Method\,2} = \frac{\Pr(\mathrm{A})}{\Pr(\mathrm{A}) + \Pr(\mathrm{B}) + \Pr(\mathrm{C})} > \frac{\Pr(\mathrm{A})}{\Pr(\mathrm{A}) + \Pr(\mathrm{B}) + \Pr(\mathrm{C}) + \Pr(\mathrm{D}) + \Pr(\mathrm{E})} = \lambda_1 \tag{7}$$

Here, we calculate the hazard ratio at each time-zero in method 2. From (7), the following inequality holds.

$$\frac{\lambda_{2trt}^{Method\ 2}}{\lambda_{1non}^{Method\ 2}} \leq \frac{\lambda_3}{\lambda_1} = \frac{\mathrm{h}(2)\exp(\beta)}{\mathrm{h}(1)} \tag{8}$$

In this case, the hazard ratio may become either larger or smaller than the true value $\exp(\beta)$, depending on the baseline hazard. Although the hazard in the untreated group tends to be overestimated, a bias arises due to the misalignment of time-zero. If the baseline hazard is constant over time, the hazard ratio will be smaller than $\exp(\beta)$. Due to the influence of immortal time bias, the value of $\frac{\lambda_3}{\lambda_1}$ itself is underestimated. However, when the baseline hazard differs over time, this value does not correspond to the true hazard ratio. In the excluded approach, in addition to immortal time bias, bias may also arise from comparing different time points.

For method 3, the hazards are:

$$\lambda_{2trt}^{Method\ 3} = \frac{\Pr(D)}{\Pr(D) + \Pr(E)} = \lambda_3 \tag{9}$$

$$\lambda_{2non}^{Method\ 3} = \frac{\Pr(B)}{\Pr(B) + \Pr(C)} = \lambda_2 \tag{10}$$

Method 3 is the appropriate method, as $\lambda_{2trt}^{Method\ 3}$ and $\lambda_{2non}^{Method\ 3}$ correctly equal to the true hazard.

In summary, under method 1, if no individuals have initiated treatment at the CED time point, the hazard ratio becomes zero until treatment begins. In method 2, the hazard ratio may be biased either upward or downward due to the influence of the baseline hazard. However, if the baseline hazard remains constant over time, the bias will tend to be downward. In contrast, under method 3, no bias arises under the current setting.

eMaterial 2. Simulation study in the presence of time-varying confounding

Here, we conduct a simulation study to quantify the magnitudes of biases in methods 1 and 2 in the presence of a time-varying confounder.

Setting

The simulation was conducted with reference to Young et al. (2014).[1] The sample size was set at n=10,000 with 10 time points and the simulation was repeated 1,000 times.

For each subject $i = 1, \cdots, n$ and each time point $t = 1, \cdots, 10$, data are generated according to the following steps:

First, generate $L_{i,1}$ from a Bernoulli distribution with the following probability:

$$\Pr[L_{i,1} = 1] = 0.5 \quad (1)$$

Next, generate the treatment at the first time point from a Bernoulli distribution with the following probability:

$$\Pr[A_{i,1} = 1 | L_{i,1} = l_{i,1}] = \frac{\exp(\alpha_0 + \alpha_1 l_{i,1})}{1 + \exp(\alpha_0 + \alpha_1 l_{i,1})} \quad (2)$$

For $t = 2, \cdots, 10$, generate the variables according to the following procedure.

1. Outcome $Y_{i,t}$

   The outcome variable $Y_{i,t}$ is generated from a Bernoulli distribution with probability given by equation (3):

$$\Pr[Y_{i,t} = 1 | \bar{L}_{i,t-1} = \bar{l}_{i,t-1}, \bar{A}_{i,t-1} = \bar{a}_{i,t-1}, Y_{i,t-1} = 0] = \frac{\exp(\theta_0 + \theta_1 l_{i,t-1} + \theta_2 a_{i,t-1} + \theta_3 a_{i,t-2})}{1 + \exp(\theta_0 + \theta_1 l_{i,t-1} + \theta_2 a_{i,t-1} + \theta_3 a_{i,t-2})} \quad (3)$$

2. Binary Covariate $L_{i,t}$

The binary covariate $L_{i,t}$ is generated from a Bernoulli distribution with probability given by equation (4):

$$\Pr\left[L_{i,t} = 1 \middle| \bar{L}_{i,t-1} = \bar{l}_{i,t-1}, \bar{A}_{i,t-1} = \bar{a}_{i,t-1}, Y_{i,t} = 0\right] = \frac{\exp\left(\beta_1 a_{i,t-1}\right)}{1 + \exp\left(\beta_1 a_{i,t-1}\right)} \tag{4}$$

3. Binary Treatment Variable $A_{i,t}$

The binary treatment variable $A_{i,t}$ is generated with probability given by equation (5).

$$\Pr\left[A_{i,t} = 1 \middle| \bar{L}_{i,t} = \bar{l}_{i,t}, \bar{A}_{i,t-1} = \bar{a}_{i,t-1}, Y_{i,t} = 0\right] = (1 - a_{i,t-1}) \frac{\exp\left(\alpha_0 + \alpha_1 l_{i,t}\right)}{1 + \exp\left(\alpha_0 + \alpha_1 l_{i,t}\right)} + a_{i,t-1} \tag{5}$$

Specifically, if $A_{i,t-1} = 0$, then the treatment probability follows a Bernoulli distribution with:

$$\frac{\exp\left(\alpha_0 + \alpha_1 l_{i,t}\right)}{1 + \exp\left(\alpha_0 + \alpha_1 l_{i,t}\right)}$$

If $A_{i,t-1} = 1$, then $A_{i,t}$ is deterministically set to 1.

Here, we consider the following marginal structural Cox model. $\bar{a}$ represent the history of treatment from the beginning to the end.

$$\lambda_T^{\bar{a}}(t) = \lambda_{T^0}(t) \exp\left(\psi_o a_t + \psi_1 a_{t-1}\right)$$

Under the assumption of rare events, the following equation holds: $\psi_0 = \theta_2$, $\psi_1 = \log\left\{\frac{2\exp(\theta_3)\{1+\exp(\beta_1+\theta_1)\}}{\{1+\exp(\beta_1)\}\{1+\exp(\theta_1)\}}\right\}$[1]. We set both ψ_0 and ψ_1 to 0, meaning $\theta_2 = 0$ and $\theta_3 = \log\left\{\frac{\{1+\exp(\beta_1)\}\{1+\exp(\theta_1)\}}{2\{1+\exp(\beta_1+\theta_1)\}}\right\}$. Under this scenario, the true hazard ratio is 1. The parameter values used in this simulation are: $\beta_1 = 0.5$, $\alpha_0 = -5$, $\alpha_1 = 2$, $\theta_0 = -3.5$, $\theta_1 = -1.5$, $\theta_2 = 0$, $\theta_3 = \log\left\{\frac{\{1+\exp(\beta_1)\}\{1+\exp(\theta_1)\}}{2\{1+\exp(\beta_1+\theta_1)\}}\right\} \cong 0.17$. In this setting, the average proportion of individuals starting to receive treatment at each time point was approximately 2.7%, while the proportion developing the

outcome at each time point at risk was approximately 2%. To confirm the impact of reducing the treatment proportion, the analysis was also conducted with $\alpha_0 = -7$, where the proportion of individuals starting to receive treatment at each time point was approximately 0.4%. To examine the impact of reversing the direction of confounding, we conducted an additional analysis by reversing the sign of α_1. $\beta_1 = 0.5$, $\alpha_0 = -5$, $\alpha_1 = -2$, $\theta_0 = -3.5$, $\theta_1 = -1.5$, $\theta_2 = 0$, $\theta_3 = \log\left\{\frac{\{1+\exp(\beta_1)\}\{1+\exp(\theta_1)\}}{2\{1+\exp(\beta_1+\theta_1)\}}\right\} \cong 0.17$.

Statistical analysis

We compare three time-zero settings: methods 1, 2, and 3. Follow-up is conducted from time-zero to the occurrence of the outcome or the end of the study. At the time-zero for each method, 1:1 propensity matching is performed. We estimate the hazard ratio (HR) using a Cox regression model to assess the intention-to-treat (ITT) effect. For comparison, we also conduct a time-dependent Cox regression including time-varying treatment and confounder as explanatory variables.

Simulation results

Figure 1 shows the simulation results. When comparing the three time-zero settings using non-users as the control group, we found that in method 1, the hazard ratio estimates were negatively biased from the true value of 1, resulting in an overestimation of the treatment effect. Similarly, in method 2, the hazard ratio estimates were also negatively biased from the true value. Even when the treatment proportion was further reduced, the magnitude of bias remained largely unchanged (**Figure 1**). Adjusting for time-varying confounders using Cox regression resulted in positive bias, whereas method 3 showed almost no bias. When the direction of confounding was reversed, Cox regression produced negatively biased results. However, the relative trends among methods 1 to 3 remained consistent (**Figure 2**). Finally, we conducted a Cox regression analysis under the assumption that no

time-varying confounding was present, using the same setting as in the main text with a 27 % treatment proportion. In this scenario without time-varying confounding, Cox regression provided nearly unbiased estimates (**Figure 3**).

REFERENCES

1 Young JG, Tchetgen Tchetgen EJ. Simulation from a known Cox MSM using standard parametric models for the g-formula. Stat Med 2014;33:1001–14.

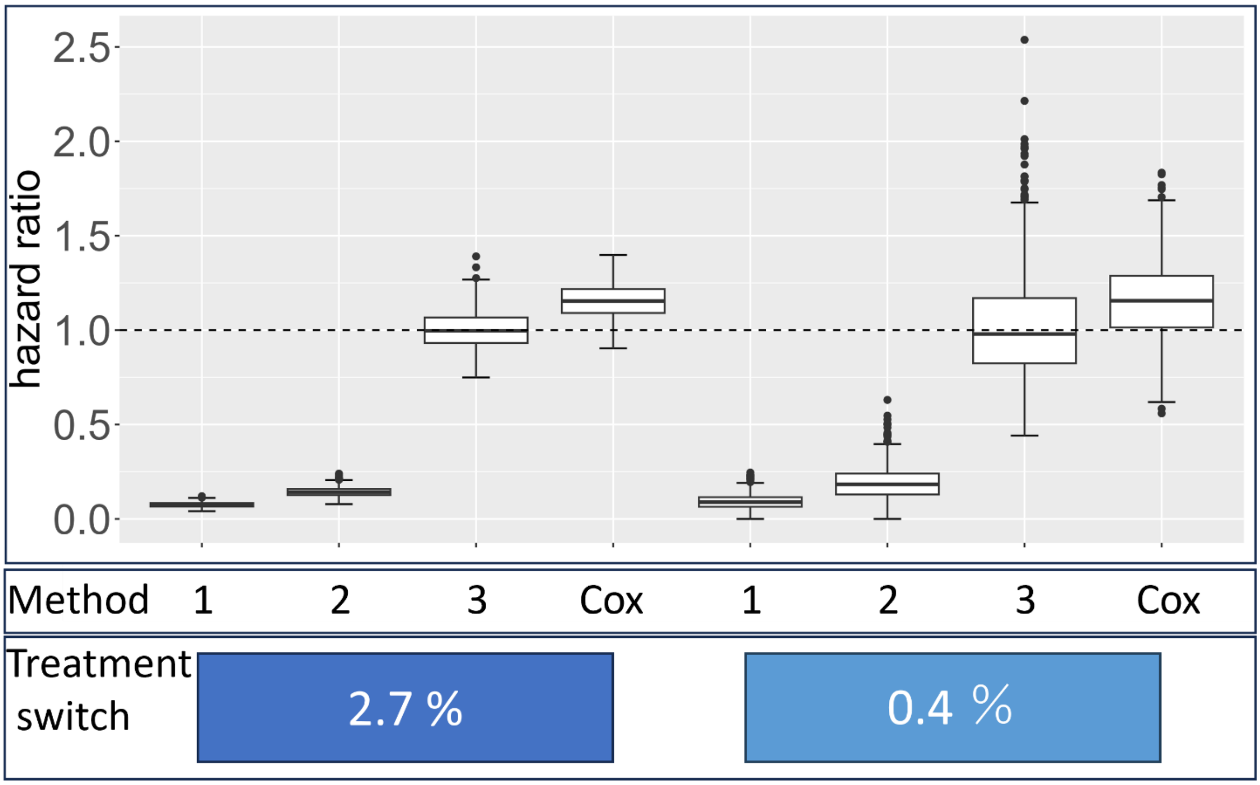

eFigure 1. Simulation results comparing methods 1, 2, 3 and time-dependent Cox regressions adjusted for time-varying confounders. The figure shows box plots of estimated hazard ratios over 1,000 simulation runs, with the true value set at 1. The average proportion of individuals receiving treatment at each time point was approximately 2.7 % or 0.4 %, while the proportion developing the outcome was approximately 2 %.

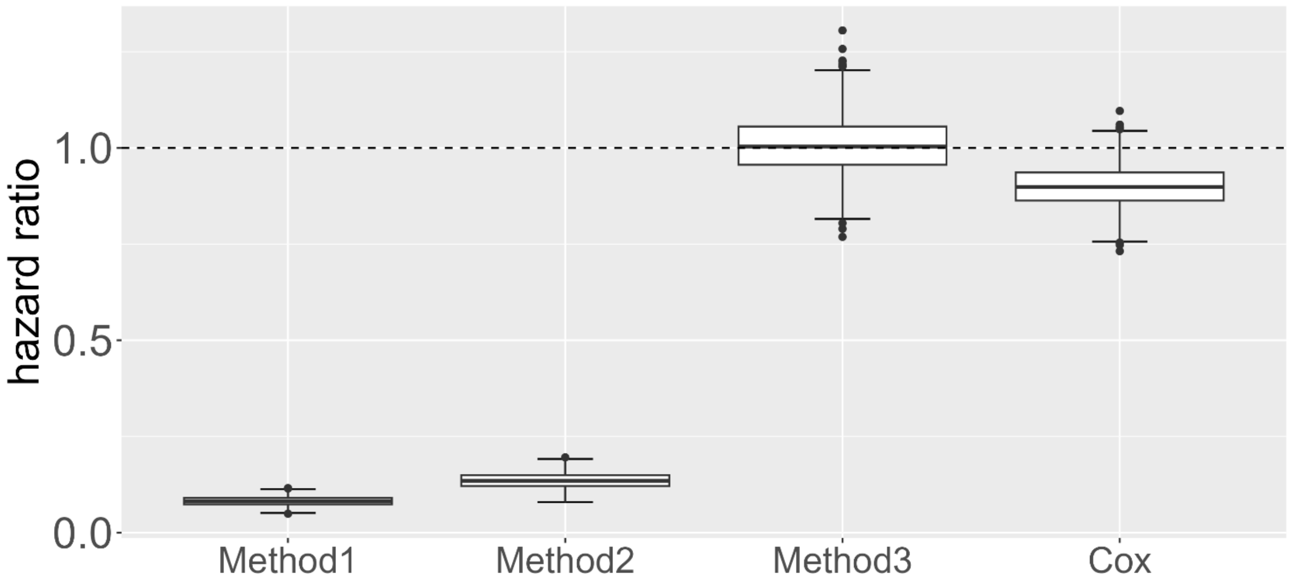

eFigure 2. Simulation results comparing methods 1, 2, 3 and time-dependent Cox regressions adjusted for time-varying confounders. The figure shows box plots of estimated hazard ratios over 1,000 simulation runs, with the true value set at 1. The average proportion of individuals receiving treatment at each time point was approximately 2.7 %, while the proportion developing the outcome was approximately 2 %.

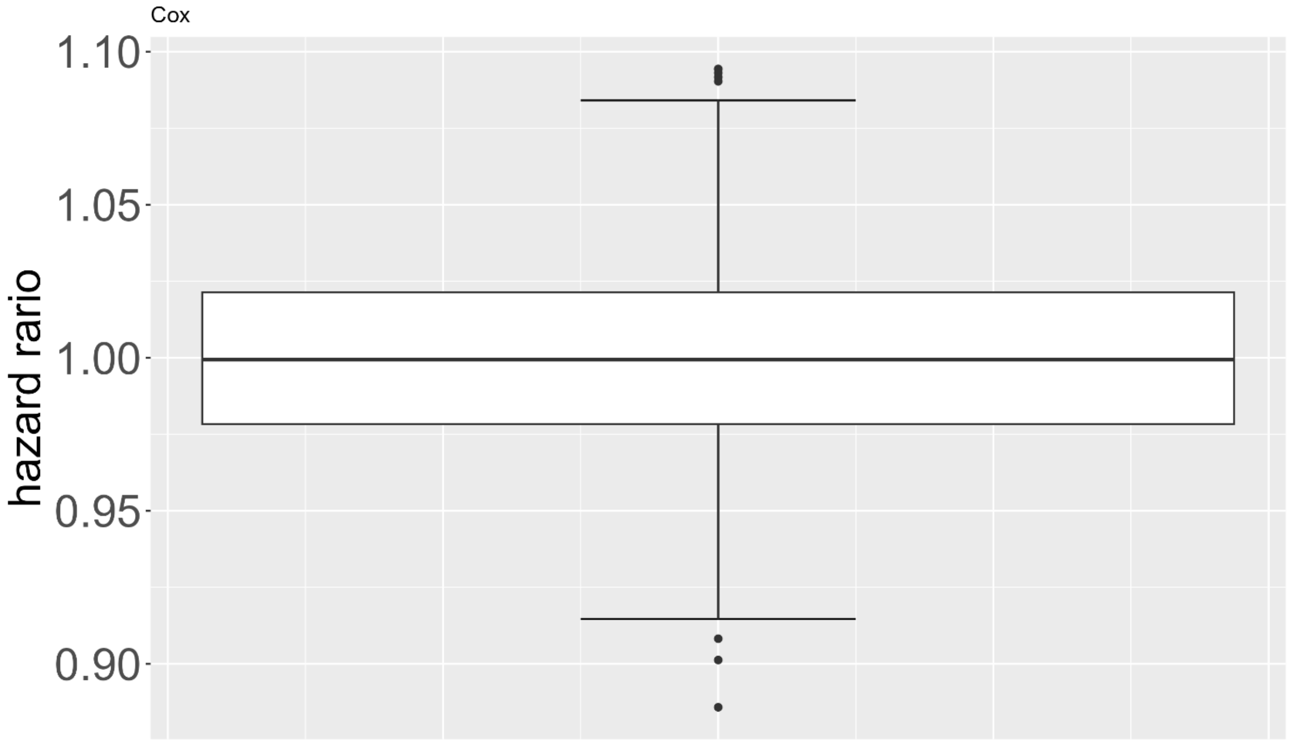

eFigure 3. Simulation results of Cox regression without time-varying confounders. The figure shows box plots of estimated hazard ratios over 1,000 simulation runs, with the true value set at 1. The probabilities of receiving treatment at each time point are set at 27 %.
